# Transparent Body-Attachable Multifunctional Pressure, Thermal, and Proximity Sensor and Heater

**DOI:** 10.1038/s41598-020-59450-0

**Published:** 2020-02-14

**Authors:** Hong Seok Jo, Seongpil An, Hyuk-Jin Kwon, Alexander L. Yarin, Sam S. Yoon

**Affiliations:** 1 School of Mechanical Engineering, Korea University, Seoul, 02841 Republic of Korea; 2 SKKU Advanced Institute of Nanotechnology (SAINT) and Department of Nano Engineering, Sungkyunkwan University (SKKU), Suwon, 16419 Republic of Korea; 3 Department of Mechanical and Industrial Engineering, University of Illinois at Chicago, 842 W. Taylor Street, Chicago, Illinois 60607-7022 United States

**Keywords:** Quality of life, Electrical and electronic engineering

## Abstract

A multifunctional sensor capable of simultaneous sensing of temperature, pressure, and proximity has been developed. This transparent and body-attachable device is also capable of providing heat under low voltage. The multi-sensor consists of metal fibers fabricated by electrospinning and electroplating. The device comprises randomly deposited metal fibers, which not only provide heating but also perform as thermal and proximity sensors, and orderly aligned metal fibers that act as a pressure sensor. The sensor is fabricated by weaving straight rectangular electrodes on a transparent substrate (a matrix). The sensitivity is readily enhanced by installing numerous matrices that facilitate higher sensing resolution. The convective heat transfer coefficient of the heater is *h* = 0.014 W·cm^−2^·°C^−1^. The temperature coefficient of resistivity (TCR) and pressure sensitivity (*η*_P_) are 0.038 °C^−1^ and 5.3 × 10^−3^ kPa^−1^, respectively. The superior sensitivity of the device is confirmed via quantitative comparison with similar devices. This multifunctional device also has a superior convective heat transfer coefficient than do other heaters reported in the literature.

## Introduction

Recently, a new reality known as the fourth industrial revolution (4IR), representing the coalescence of digital (or cyber) systems and hardware technology, has come into being and has already begun to play a central role in our daily lives^1^. The exponential technological growth during the 4IR has led to development of various systems, such as artificial electronic skin (e-skin), a transparent and flexible material capable of sensing external stimuli akin to the human somatosensory system^2–4^. Such electronic skins could allow one to cyber-physically interact with the environment via changes in temperature, humidity, infrared light intensity, *etc*^5,6^. In the near future, e-skin-laden clothing is expected to facilitate not only the acquisition of information about one’s surroundings but also monitoring and digitalization of an individual’s physical conditions. This technology is thus expected to contribute to the significant advancement in the global healthcare system, as well as the global textile and clothing industry.

Indeed, the advent of the 4IR has recently led to numerous studies on multifunctional sensors possessing transparency and flexibility to meet the demand of e-skin applications^7^. Nevertheless, it should be emphasized that most prior multifunctional sensors are restricted to only single or double functionality, designed for sensing pressure^8,9^, temperature^10,11^, proximity^4,12^, or pressure-temperature combinations^3,13^. Even though several recent studies have reported triple-functional or over-triple-functional sensors developed by applying various nanomaterials and fabrication techniques^6,14–16^. Such sensors generally suffer from issues such as severe restrictions in transparency and efficiency of the fabrication process, which hinder their use as a practical and human-friendly e-skin.

For example, Hua *et al*. recently introduced a highly stretchable multifunctional sensor with the capacity for monitoring pressure, temperature, proximity, humidity, light intensity, in-plane strain, and magnetic fields by using various materials and methods^15^. Despite its multifunctionality, the highly complex and time-consuming multistep manufacturing requirements not only lessened the potential for low-cost, large-scale production^17,18^, but also precluded the achievement of high transparency for these sensors. The multilayers made the sensor opaque and non-transparent, which can hinder their use as supplementary electronics in e-textile applications^19,20^. Transparent sensors are aesthetically advantageous because they can be attached to e-textile substrates without being noticed. In addition, because multifunctional sensors for e-skins require some degree of transparency, a new approach to achieve transparency and flexibility with a facile fabrication process is required.

The present group recently developed highly conductive and ultra-thin metal-plated nanofibers (NFs) having a unique percolative network via electrospinning and electroplating. These electrospun and electroplated metal fibers are stretchable, flexible, and highly conductive and thus act as excellent transparent conducting electrode (TCE) materials^4,21–23^. These metal fibers are superior to other existing materials intended for similar purposes in terms of their sheet resistance (*R*_s_) and transparency^21^. The high purity of the metal NFs enables the achievement of an extremely low *R*_s_ for the TCE film, while the percolative structure with openings (between NFs) facilitates high transparency.

Herein, metal fibers are weaved to produce a highly transparent and flexible e-skin multisensor for simultaneous monitoring of pressure, temperature, and proximity that can also function as a heater. To the best of our knowledge, this type of multisensor with superior transparency has not yet been demonstrated. Furthermore, the proposed method of fabrication is simple, low-cost, and thus economically viable. The fabrication methods introduced herein include four simple steps, namely, electrospinning, electroplating, bar-coating, and transfer. Furthermore, the metal of choice is nickel, which is highly resistant to corrosion and thus affords long-term durability. Existing multifunctional sensors are capable of passively sensing several external stimuli, whereas the sensor developed herein is capable of passive and also active control, so that it holds great promise as a next-generation wearable transparent multifunctional sensor for e-skin.

## Experimental Section

### Materials

Polymer NFs were obtained by electrospinning a 8 wt% polyacrylonitrile (PAN, *M*_w_ = 150 kDa, Sigma-Aldrich, USA) solution using *N*,*N*-dimethylformamide (DMF, 99.8%, Sigma-Aldrich, USA) as the solvent. The aqueous nickel (Ni) electroplating solution was prepared by dissolving 6 g of H_3_BO_3_ (Sigma-Aldrich, USA) and 80 g of Ni(SO_3_NH_2_)_2_·4H_2_O (Sigma-Aldrich, USA) in 200 mL of DI water; 1 M NaOH (Sigma-Aldrich, USA) was added to adjust the pH of the electroplating solution to 4.5. Then, the nickel electroplating solution was magnetically stirred for 3 h at 35 °C on a hot plate. A polydimethylsiloxane (PDMS) plate was prepared by mixing a silicone elastomer (01064291, DOW CORNING, USA) and a curing agent (01015311, DOW CORNING, USA) in a 10:1 ratio. The silicone bonder had a viscosity of *μ* = 15 Pa·s, density of *ρ* = 1.04 g/cm^2^, tensile strength of 1.7 MPa, and thermal conductivity of *k* = 0.21 Wm^−1^k^−1^.

### Fabrication of non-aligned and aligned Ni fibers

The fabrication processes of non-aligned and aligned Ni fibers (Ni Fs) involved a combination of electrospinning and electroplating, as illustrated in Fig. S1, where different types of collectors were employed in the electrospinning process to control the alignment of the electrospun PAN NFs (cf. Fig. S1a,b). In electrospinning, solidified NFs are deposited on a planar collecting electrode randomly because they undergo multi-loop bending instability triggered by random perturbations^24,25^. Conversely, sharpened, two-pinned collectors contract the electric field line, which facilitates the delivery of NFs to specific locations and their alignment^25,26^.

For the non-aligned Ni Fs, PAN NFs were electrospun onto a flat collector (Fig. S1a); the distance between the needle (25 gauge, EFD) and the collector was 13 cm, and the electrospinning time was *t*_es_ = 2 s. The flow rate of the 8 wt% PAN solution was fixed at 200 μL·h^−1^ using a syringe pump (Legato 100, KD Scientific). The applied voltage to the electrospinning the PAN solution was set at 5 kV, provided by a DC high-voltage supply (EL20P2, Glassman High Voltage, USA). The non-aligned electrospun PAN NFs were transferred to a square copper (Cu) frame (3 × 2 cm^2^, cf. Fig. S1c). Thereafter, Pt seeds were deposited on the NFs by sputtering (Vacuum Device Inc., MSP-1S) for 2.5 s using a current of 40 mA. The Pt-seeded PAN NFs were electroplated with Ni for various electroplating times (*t*_ep_ = 40, 60, 80, and 100 s) at 6 V (using an E3642A 50 W power supply, Agilent, USA). Finally, the Ni-plated fibers were fully dried by blowing with N_2_ gas for a few minutes.

In contrast with the non-aligned Ni Fs, the aligned Ni Fs were deposited by electrospinning PAN NFs onto a two-pinned collector for a *t*_es_ of 100 s (where the height and length of each pin were 3 cm and 7 cm, respectively, and the distance between the two pins was 5 cm, cf. Fig. S1b). The flow rate and the applied voltage were identical to those used for electrospinning of the non-aligned Ni Fs. The transfer, electroplating, Pt-seeding, and drying processes of the aligned fibers were also identical to those used for the non-aligned Ni Fs; however, the electrospinning time *t*_ep_ differed (*t*_ep_ = 40, 80, 100, and 150 s for the aligned Ni Fs).

### Fabrication of heater and sensors, and assembly of multifunctional sensor

**Figure 1:**
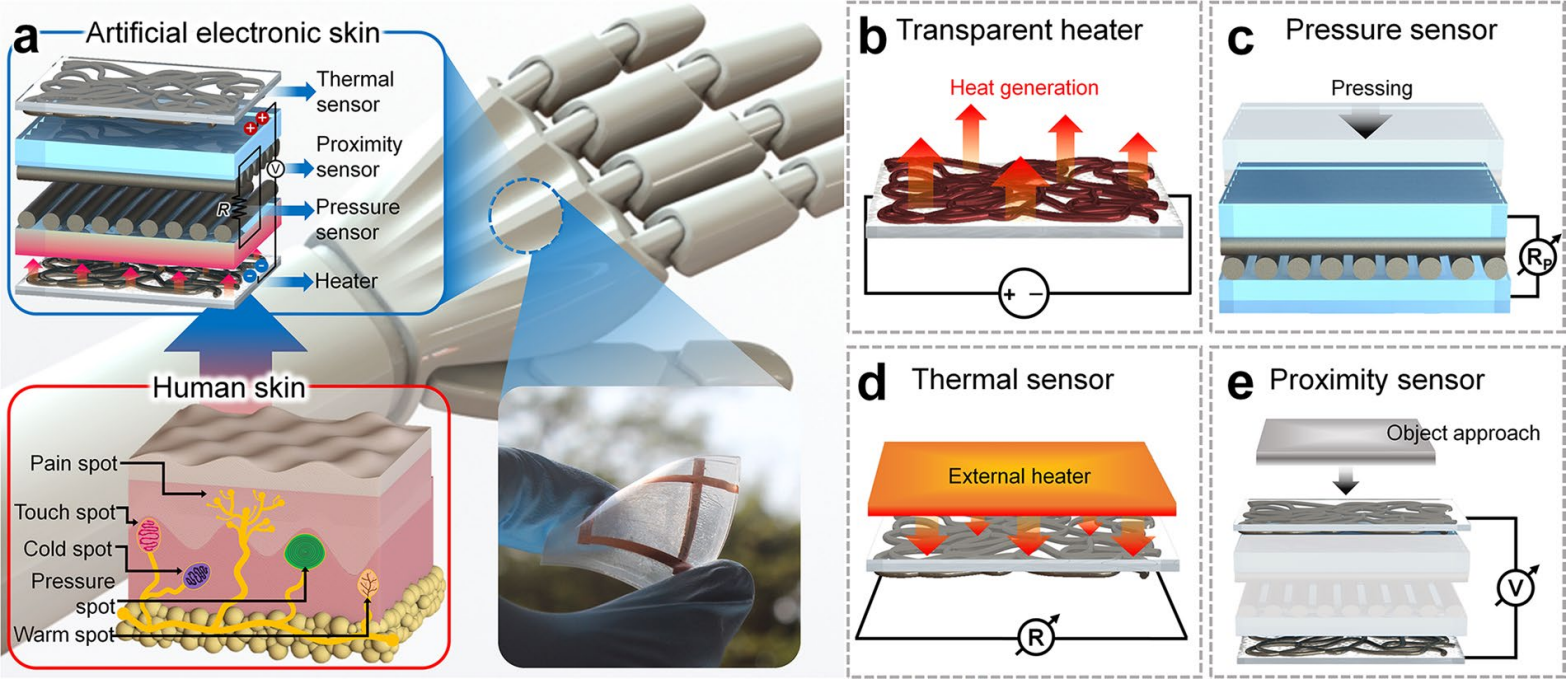
Illustration of multifunctional sensor for flexible artificial electronic skin. (**a**) Enlarged view of stacked layers of the multifunctional sensor and comparison with human subcutaneous tissue. Multifunctional sensing performance: (**b**) heat generation and (**c**) pressure, (**d**) thermal, and (**e**) proximity sensing.

The cutaneous sensation in human skin is controlled by pain, touch, pressure, and cold/warm receptors (or spots), as illustrated in Fig. 1a, which demonstrates how these sensations can be simultaneously perceived by the nervous system. Inspired by the fact that the subcutaneous sensory receptors are separated (or layered), the multifunctional sensor was designed to detect different stimuli in different layers.

The multifunctional sensor developed in this study consists of four layers, as depicted in Fig. 1a. The bottom-most layer of the sensor is a transparent heater composed of non-aligned Ni Fs that can generate heat as a result of Joule heating (Fig. 1b). The second and third layers from the bottom are composed of aligned Ni Fs, where a particular pressure can be detected when the second and third layers come into close contact, as depicted in Fig. 1c. The top layer, composed of non-aligned Ni Fs, is designed as a thermal sensor, where the underlying working mechanism is based on the linear variation of the resistance of the metal, which depends on the temperature change (Fig. 1d). Finally, measurement of the voltage between the bottom and top layers enables the detection of an approaching object, thus enabling the device to function as a proximity sensor (Fig. 1e). The outputs of each sensor, e.g., voltage and resistance, are measured independently.

**Figure 2:**
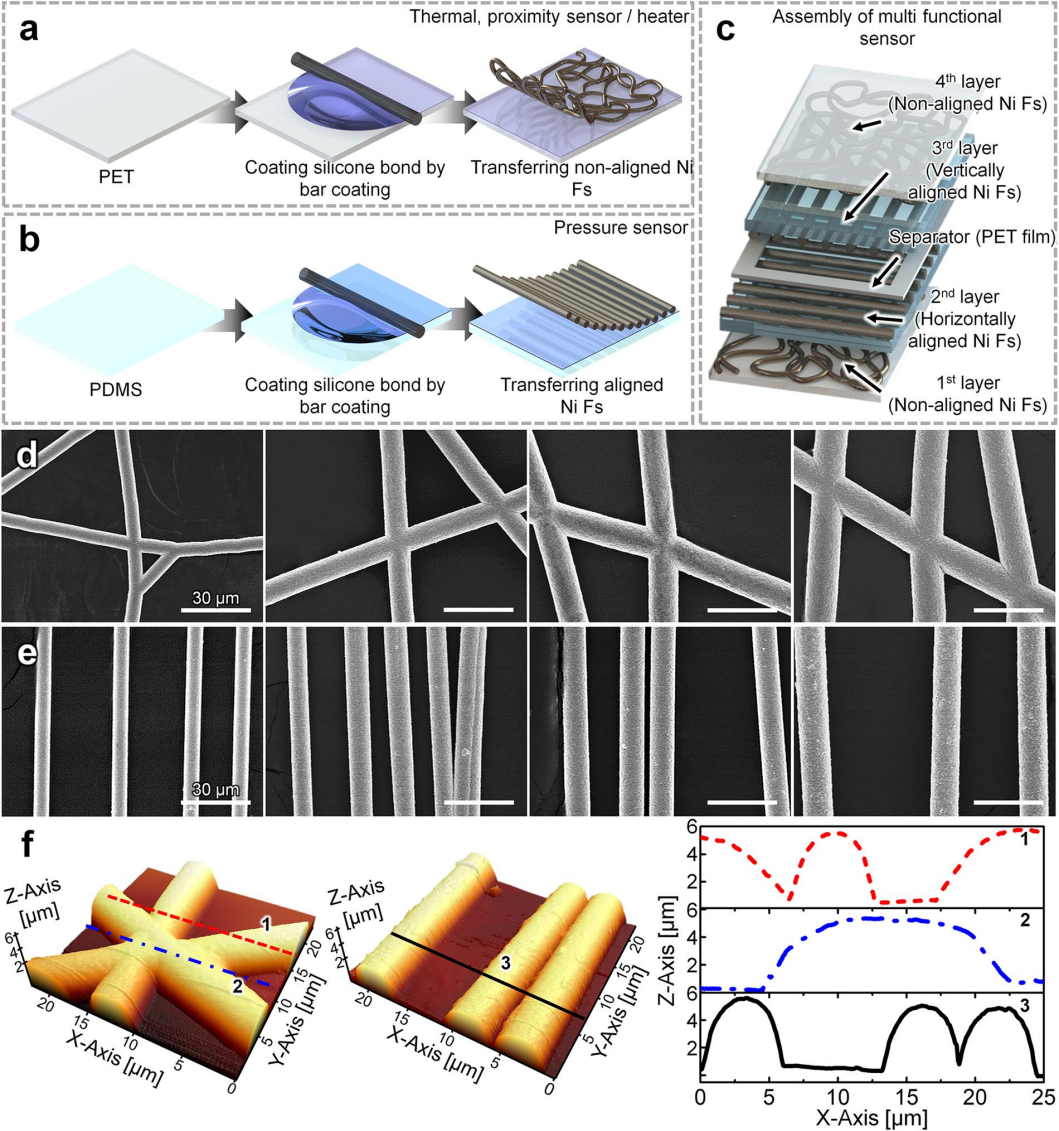
Process of fabrication of (**a**) thermal sensor, proximity sensor, and transparent heater based on non-aligned Ni Fs, and (**b**) pressure sensor based on aligned Ni Fs. (**c**) Assembly of the thermal, proximity, and pressure sensors, and a transparent heater. Top-view SEM images of (**d**) non-aligned Ni Fs with *t*_es_ = 2 s as *t*_ep_ increased from 40 to 100 s (from left to right), and (**e**) aligned Ni Fs with *t*_es_ = 100 s as *t*_ep_ increased from 40 to 150 s (from left to right). (**f**) AFM images of the non-aligned (left) and aligned Ni Fs (middle) with *t*_ep_ = 40 s for both, and the corresponding scanned heights of the non-aligned and aligned Ni Fs along lines 1, 2, and 3 (right).

Figure 2a depicts the fabrication process of the thermal and proximity sensors, as well as the transparent heater, by employing non-aligned Ni Fs. First, a silicone bonder was deposited on a PET film (4 × 4 × 0.02 cm^3^) using a bar coater (RDS #2, RDS, USA). Thereafter, the non-aligned Ni Fs (2 × 2 cm^2^) were transferred onto the silicone bonder-coated PET film. Note that the fabrication process of the pressure sensor is identical to those used for the above-mentioned sensors, except that a PDMS film was used instead of a PET film (4 × 4 × 0.1 cm^3^), and aligned Ni Fs were used (Fig. 2b).

The assembly of the multifunctional sensor is shown in Fig. 2c. First, the PET film with non-aligned Ni Fs was arranged as the bottom layer (or the first layer) of the multifunctional sensor. Then, PDMS films with aligned Ni Fs, corresponding to the second and the third layers, were superposed onto the first layer. The orientations of the aligned Ni Fs in the second and the third layers were perpendicular to each other, and a frame-like polyester (PET) separator (1.8 × 1.8 × 0.02 cm^3^) was positioned between the second and third layers. Finally, the PET film with non-aligned Ni Fs was positioned as the topmost layer of the sensor. The layers were attached to each other using a silicone bonder.

### Characterization

The morphologies of the non-aligned and aligned Ni Fs were determined using scanning electron microscopy (SEM, S-5000, Hitachi, Japan). The transmittance (*T*) was measured using an ultraviolet-visible spectrophotometer (Optizen POP, Mecasys, Republic of Korea). The electrical properties of the multifunctional sensor, including resistance (*R*), voltage (*V*), and sheet resistance (*R*_s_), were measured using a source meter (2401 SMU, Keithley, USA) and a sheet-resistance meter (FPP-400, Dasol Eng., Republic of Korea) with a four-point probe. The surface temperature (*T*_s_) of the heater and preheater, used for evaluating the performance as a thermal sensor, were recorded using a data recorder (Hioki LR8400-20, HIOKI, Japan) with a thermocouple and an infrared (IR) camera (FLIR-E63900, FLIR, Wilsonville, OR, USA) with an emissivity of *ε* = 0.95, which corresponds to the emissivity of the PET film.

## Results and Discussion

### Non-aligned and aligned Ni Fs

Figure 2d shows SEM images of the non-aligned Ni Fs at various electroplating times (*t*_ep_ = 40–100 s); *t*_es_ was fixed at 2 s (cf. Experimental section). The average diameter (*D*) of the non-aligned Ni Fs gradually increased from 6.18 to 10.3, 13.2, and 14.7 μm as the electroplating time *t*_ep_ increased from 40 to 60, 80, and 100 s, respectively. This is because the amount of Ni accumulated over the surface of PAN NFs increased as *t*_ep_ increased.

The transmittance (*T*), resistance (*R*), and sheet resistance (*R*_s_) of the non-aligned Ni Fs with increasing *t*_ep_ (which also contributes to the increase in *D*) are listed in Table 1. The transmittance of the non-aligned Ni Fs decreased from 92.9% to 88.7%, 83.1%, and 78.4% (cf. Fig. S2a) as *t*_ep_ increased because *D* increased (cf. Fig. S2d). In addition, *R* decreased from 1.52 to 0.78, 0.50, and 0.28 Ω as *t*_ep_ increased, and the corresponding *R*_s_ value also decreased from 0.638 to 0.331, 0.142, and 0.107 Ω sq^−1^, where the improvement in the conductivity is attributed to the increase in *D* (see Table 1). Notably, in contrast with the trends in *T*, *R*, and *R*_s_ as a function of *t*_ep_, the highest figure-of-merit (FoM) of 1.106 Ω^−1^ was achieved with *t*_ep_ = 80 s, where, FoM = *T*^10^/*R*_s_. When the time *t*_ep_ was increased from 40 to 100 s, the *R*_s_ ratio decreased by 48.1% (from *t*_ep_ = 40 to 60 s), 57.1% (from *t*_ep_ = 60 to 80 s), and 24.6% (from *t*_ep_ = 80 to 100 s). Accordingly, the FoM at *t*_es_ = 80 s was the highest because the *R*_s_ decreased most steeply between *t*_ep_ = 60 and 80 s, while *T* decreased by 5.6% (from *T* = 88.7 to 83.1%).

**Table 1:** Properties of the non-aligned and aligned Ni Fs.

	*t*_es_ (s)	*t*_ep_ (s)	*D* (μm)	*A* (cm^2^)	*R*_0_ (Ω)	*R*_s_ (Ω sq^−1^)	*T* (%)	FoM (Ω^−1^)
Non-aligned Ni Fs	2	40	6.18	1.63	1.52	0.638	92.9	0.750
		60	10.3	2.72	0.68	0.331	88.7	0.911
		80	13.2	3.48	0.40	0.142	83.1	1.106
		100	14.7	3.88	0.28	0.107	78.4	0.819
	***t***_**es**_ **(s)**	***t***_**ep**_ **(s)**	***D*** **(μm)**	***R***_**0**_ **(Ω)**		***R***_**s**_ **(Ω sq**^**−1**^**)**	***T*** **(%)**	**FoM (Ω**^**−1**^**)**
Aligned Ni Fs	100	40	6.2	0.98		1.99	90.8	0.191
		60	10.1	0.56		0.81	86.6	0.293
		100	12.2	0.33		0.52	81.9	0.261
		150	14.2	0.15		0.35	77.9	0.235

The average diameter, *D*, of the non-aligned Ni Fs affected the values of *T*, *R*, and *R*_s_ of these fibers, as well as the surface area (*A*) of the non-aligned Ni Fs. Note that for each case, the length per unit area of the non-aligned Ni Fs (cf. Fig. 2d) was obtained by investigating ten SEM images, which yielded almost the same value of 0.021 μm∙μm^−2^ because the electrospinning time *t*_es_ was the same (*t*_es_ = 2 s; cf. Experimental section) for all cases. Accordingly, the total length (*L*) of the non-aligned Ni Fs over an area of 4 cm^2^ was 8.4 m (= 0.021 μm μm^−2^ × 4 cm^2^). With an increase in the electroplating time *t*_ep_, the surface area of the non-aligned Ni Fs, defined as *A* = π*DL*, over the area of 4 cm^2^ increased to 1.63, 2.72, 3.48, and 3.88 cm^2^, respectively (see Table 1).

As revealed in Fig. 2e, the diameter *D* of the aligned Ni Fs increased from 6.2 to 10.3, 13.2, and 14.7 μm as the electroplating time *t*_ep_ increased from 40 to 60, 100, and 150 s, respectively, where the electrospinning time *t*_es_ was fixed at 100 s. Similar to the case of the non-aligned Ni Fs, *R*, *R*_s_, and *T* for the aligned Ni Fs were influenced by the change in *D*. Here, the *R* value was measured using electrodes attached to both ends of the aligned Ni Fs, whereas the *R*_s_ value was measured using a sheet resistance meter (cf. Experimental section). The *R* value of the aligned Ni Fs decreased from 0.98 to 0.15 Ω as *t*_ep_ increased, and the corresponding *R*_s_ decreased from 1.99 to 0.35 Ω sq^−1^ with increasing *D* (Table 1). In addition, *T* of the aligned Ni Fs gradually decreased from 90.8% to 86.6%, 81.9%, and 77.9% with increasing *t*_ep_ (cf. Fig. S2b,e). The FoM increased from 0.191 to 0.293 Ω^−1^ as *t*_ep_ increased from 40 to 60 s because the corresponding *R*_s_ decreased dramatically, whereas the FoM slightly decreased from 0.261 to 0.235 Ω^−1^ as *t*_ep_ increased from 100 to 150 s because of the moderate decrease in *R*_s_. The non-aligned fibers are randomly deposited and have a higher number of junctions, which are fused. Accordingly, their sheet resistance (*R*_s_) is relatively lower. The aligned fibers are not prone to yielding many fused junctions; thus, their sheet resistance is relatively higher; see Table 1. As a result, the FoM of the non-aligned fibers is higher than that of the aligned fibers.

It should be emphasized that the height at the junction of the non-aligned Ni Fs (*t*_ep_ = 40 s) was similar to that of a single Ni F, as shown in Fig. 2f, where the scanned heights over lines 1 and 2 were almost the same (0.58 μm). Similarly, for the aligned Ni Fs, no discernible variation in the heights of different aligned Ni Fs (*t*_ep_ = 40 s) was observed; the corresponding height of the aligned Ni Fs was also 0.58 μm (Fig. 2f). Such uniform roughness of the non-aligned and aligned Ni Fs holds great promise for their potential use as industrially feasible TCEs for touch screens, organic light-emitting diodes, solar cells, *etc*^27–29^.

Nevertheless, the aligned Ni Fs presented lower resistance (*R*_0_) than the non-aligned Ni Fs for the following reason. When the non-aligned Ni Fs are transferred to a substrate (cf. Fig. 2c and Experimental section), the edges of the non-aligned Ni Fs are inevitably cut, as depicted in Fig. S2f, causing an increase in the resistance because the electrons cannot readily move between different Ni Fs. In contrast, the electrons in the aligned Ni Fs can move along the parallel Ni Fs, regardless of whether they are cut or not (see Fig. S2f), which results in a higher conductivity (and accordingly, a lower resistance) for the aligned Ni Fs relative to that of the non-aligned Ni Fs (cf. Table 1). The pattern of *R*_0_ should be distinguished from the pattern of *R*_s_.

### Heating and thermal sensing performance

As an e-skin, the heater function can be useful in maintaining a comfortable temperature surrounding the body. The heater temperature can increase according to the user’s need. The heating performance of the non-aligned Ni Fs in the multifunctional sensor was explored by applying a voltage (*V*_a_) (E3642A 50 W power supply, Agilent, USA) to the sensor and varying it from 0.1 to 0.2, 0.3, and 0.4 V. The heating temperature at the heater surface (*T*_s_) was measured by a thermocouple attached to the PET film in the heater of the sensor (cf. Fig. S3a). Note that the sensor was enveloped in glass wool (*k* = 0.035 W·m^−1^·K^−1^, Saint-Gobain ISOVER) and aluminum foil to shield against ambient air flow and to prevent heat loss. Accordingly, the ambient temperature (*T*_∞_) and the initial value of *T*_s_ for all cases were 26.2 °C.

**Figure 3:**
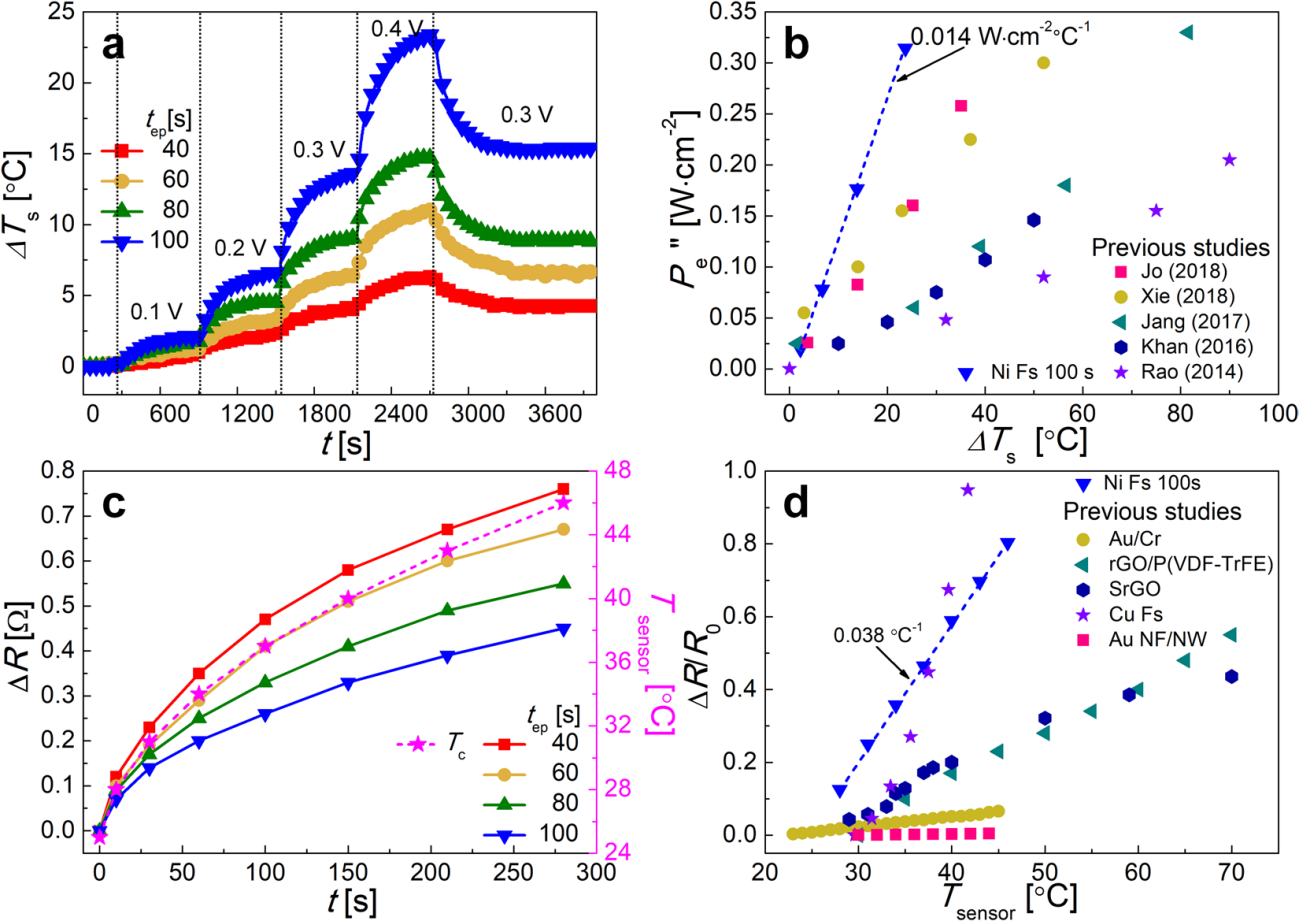
Performance of multifunctional sensor as transparent heater and thermal sensor: (**a**) surface temperature *T*_s_ of the multifunctional sensor with fibers prepared with electroplating times *t*_ep_ of 40, 60, 80, and 100 s. This temperature pattern is highly reproducible. (**b**) Comparison of heating performance of multifunctional sensor with those of previous studies. (**c**) Change in resistance of multifunctional sensor with electroplating times *t*_ep_ of 40, 60, 80, and 100 s over the course of 280 s as a function of *T*_sensor_. (**d**) Sensitivity of thermal sensing of multifunctional sensor. Slopes of lines in panel (**d**) correspond to the TCR.

As illustrated in Fig. 3a, with an increase in *V*_a_, the values of *T*_s_ increased in all cases because of Joule heating, which is related to the supplied power (*P*_e_ = *V*_a_^2^/*R* = *I*∙*V*_a_, where *I* is the current). For *t*_ep_ = 40 s (Fig. 3a), the surface temperature *ΔT*_s_ (or *T*_s_ – *T*_∞_) of the multifunctional sensor increased from 0.7 °C to 2.2 °C, 4.0 °C, and 6.3 °C, while *V*_a_ increased from 0.1 to 0.2, 0.3, and 0.4 V, respectively; see the red squares. When the electroplating time *t*_ep_ of the sensor was increased to 60, 80, and 100 s, the steady-state value of *T*_s_ of the sensor steadily increased because the corresponding *R* value decreased; *P*_e_ = *V*_a_^2^/*R*. At *V*_a_ = 0.4 V, the maximum values of *ΔT*_s_ for heaters with fibers prepared at *t*_ep_ = 40, 60, 80, and 100 s were 6.4 °C, 11.0 °C, 14.8 °C, and 23.5 °C, respectively. Furthermore, to evaluate the stability of the heating of the multifunctional sensor, the value of *V*_a_ was varied from 0.4 to 0.3 V and was maintained for 1,000 s (see Fig. 3a). Steady-state values of *ΔT*_s_ were measured in all cases, and the corresponding values of *ΔT*_s_ for the fibers prepared at *t*_ep_ = 40, 60, 80, and 100 s were 4.23 °C, 6.5 °C, 8.8 °C, and 15.4 °C, respectively (cf. Fig. 3a).

The electric power *P*_e_ was mostly converted to thermal energy as the temperature of the heater increased, while only a minimal part of *P*_e_ was lost via conduction, convection, and radiation. Hence, the power balance can be described by the following equation:

1 $${P}_{{\rm{e}}}={V}_{{\rm{a}}}I=cm\frac{d{T}_{{\rm{s}}}(t)}{dt}+{Q}_{{\rm{loss}}}$$

where *c* and *m* are the specific heat and the mass of the heater of the multifunctional sensor, respectively, and *Q*_loss_ is the out-of-plane thermal losses from the heater (the in-plane thermal losses are negligible because of the presence of a surrounding lateral insulator, as described in Fig. S3a). Note that the radiative heat losses in *Q*_loss_ can be ignored as *T*_s_ was lower than 200 °C, whereas the conductive heat losses in *Q*_loss_ should be considered because different metal-plated fibers were sequentially layered from the heater layer (see Fig. S3a). Then, the out-of-plane *Q*_loss_ can be expressed as:

2 $${Q}_{{\rm{loss}}}=hA({T}_{{\rm{s}}}-{T}_{\infty })+{h}_{{\rm{m}}}A({T}_{{\rm{s}}}-{T}_{\infty })$$

Here, *h* is the convective heat transfer coefficient at the closest surface from the heater (cf. upper direction from the heater in Fig. S3a) and *h*_m_ is the effective heat transfer coefficient at the furthest surface from the heater (which is detailed in the Supporting Information: cf. downward direction from the heater in Fig. S3a). *A* is the out-of-plane surface area of the heater.

Note that the initial value of *T*_s_ = *T*_i_. Then, *T*_s_ can be found from Eqs. (1) and (2) as:

3 $${T}_{{\rm{s}}}=\frac{{P{\prime\prime} }_{e}}{h+{h}_{{\rm{m}}}}(1-{e}^{-\frac{(h+{h}_{{\rm{m}}})A}{cm}t})+({T}_{{\rm{i}}}-{T}_{\infty }){e}^{-\frac{(h+{h}_{{\rm{m}}})A}{cm}t}+{T}_{\infty }$$

where *P*_e_″ is the power density, which is defined as *P*_e_″ = *P*_e_/*A*. The temperature *T*_i_ was equal to *T*_∞_ in the experiment. Therefore, the *T*_s_ in the steady-state is expressed as *T*_s,steady_ = *P*_e_″/(*h* + *h*_m_) + *T*_i_, and thus, *h* = *P*_e_″/(*T*_s,steady_ – *T*_i_) – *h*_m_. When *t*_ep_ was increased from 40 to 100 s, *h* increased from 0.0084 to 0.0105, 0.0119, and 0.0136 W·cm^−2^·°C^−1^, where the value of *h*_m_ is 0.0001345 W·cm^−2^·°C^−1^ (which was calculated in detail in Supporting Information), respectively (Table 2). It should be emphasized that the heating performance was enhanced as *t*_ep_ increased from 40 to 100 s. Compared with previous studies (Fig. 3b), the values of *h* obtained in the present study (*t*_ep_ = 100 s) are the highest^11,28,30–32^, which indicates that the heating performance of the non-aligned Ni Fs in the multifunctional sensor holds great promise for use of the sensor as a transparent heater itself. The heater stability is demonstrated in Fig. S3b. The sample used was the one fabricated with the electroplating time of *t*_ep_ = 100 s. At the supplied voltage of 0.3 V, *T*_s_ increased from 27 to 35.8 °C within 180 s, and this cycle was fully repeatable at least 10 times. This temperature cycle is highly periodic and reproducible^33^.

**Table 2:** Performances of the multifunctional sensor as a heater, and thermal and pressure sensors.

*t*_es_ (s)	*t*_ep_ (s)	*h* (*W cm*^*−2*^ *°C*^*−1*^)	TCR (°C^−1^)	*t*_es_ (s)	*t*_ep_ (s)	*R*_P0_ (Ω)	*η*_p_ (10^−3^·kPa^−1^)
2	40	0.0084	0.019	100	40	3.8	5.3
	60	0.0105	0.029		60	2.5	4.5
	80	0.0119	0.033		100	1.2	4.1
	100	0.0136	0.038		150	0.52	2.4

The resistance of a metal changes linearly with variation of its temperature. Based on this dependence, we employed the non-aligned Ni Fs as a thermal sensor. As shown in Fig. 3c, the resistance of the thermal sensor composed of different non-aligned Ni Fs with *t*_ep_ = 40, 60, 80, and 100 s varied with the surface temperature of the thermal sensor, denoted *T*_sensor_, with *T*_sensor_ being varied from 25.2 °C to 46 °C over 280 s by the external heater and the corresponding resistance of the non-aligned Ni Fs being measured, as shown in Fig. S3b. It should be emphasized that the tested temperature range of the thermal sensor was 25.2 °C ≤ *T*_sensor_ ≤ 46 °C. However, the thermal sensor range is not limited to these temperatures.

In all the cases, the resistance trend agreed fairly well with the variation in *T*_sensor_. For *t*_ep_ = 40 s, the initial resistance of the sensor was 2.01 Ω; thereafter, the resistance increase (*ΔR*) increased from 0 to 0.76 Ω with an increase in *T*_sensor_ (Fig. 3c). Notably, as the value of *t*_ep_ of the non-aligned Ni Fs increased to 60, 80, and 100 s, the total variation in the resistance decreased slightly, to 0.67, 0.55, and 0.45 Ω, respectively. This trend is tentatively attributed to the variation of the cross-sectional diameter *D* of the non-aligned Ni Fs^34^.

That is, the variation of the total resistance was the smallest for *t*_ep_ = 40 s, where *D* was the largest for the non-aligned Ni Fs. Conversely, the lowest variation in the resistance was observed at *t*_ep_ = 100 s, where *D* was the largest.

The sensitivity of the multifunctional sensor for thermal sensing can be characterized by the temperature coefficient of resistivity (TCR), which is defined as^35–37^:

4 $${\rm{TCR}}=\frac{R-{R}_{0}}{{R}_{0}\Delta {T}_{{\rm{sensor}}}}$$

where *R*_0_ is the initial resistance of the sensor. In contrast with the variation in the resistance, TCR gradually increased to 0.019, 0.029, 0.033, and 0.038 °C^−1^ with an increase in *t*_ep_, as summarized in Table 2. Note that the resistance of the sensor gradually increased as the value of *t*_ep_ decreased from 100 to 40 s (cf. Fig. 3c), whereas *R*_0_ for the non-aligned Ni Fs in Table 1 increased dramatically. Therefore, the highest value of TCR was obtained at *t*_ep_ = 100 s (TCR = 0.038 °C^−1^), where *R*_0_ was the lowest.

The TCR values obtained in the present study are higher than those of graphene-, Au-, and Ag-based thermal sensors, as shown in Fig. 3d ^36–38^, even though the thermal conductivities of graphene (4800 W∙m^−1^∙K^−1^), Au (314 W∙m^−1^∙K^−1^), and Ag (429 W∙m^−1^∙K^−1^) are higher than that of Ni (91 W∙m^−1^∙K^−1^). Because the junctions of the micro-sized metal fibers were completely bonded, which drastically reduced the contact resistance, the thermal sensor composed of self-junctioned metal fibers may be a more effective sensor than other types. It should be reiterated that the junctions of metal fibers were completely bonded or fused (cf. Fig. 2), which resulted in lower resistance and enhanced sensitivity. It should also be noted that the TCR value of Cu Fs (0.078 °C^−1^)^11^ is higher than that of Ni Fs (0.038 °C^−1^), but nickel outperforms copper in terms of chemical stability, and thus nickel is more durable as a corrosion-resistant material^23^. In addition, to evaluate the long-term stability of the thermal sensor, the resistance was measured while increasing the temperature *T*_sensor_ from 26.5 to 58 °C with four different intermediate levels over 7500 s. As shown in Fig. S3d, the trend in the resistance change of the thermal sensor fabricated with *t*_ep_ = 100 s almost coincided with the variation of *T*_sensor_.

### Proximity and pressure sensing performance

Proximity sensors, which can detect an approaching object, are generally based on inductive, optical, ultrasonic, and capacitive potential mechanisms^4,39–42^. Among the above-listed mechanisms, capacitive proximity allows a sensor to detect both conductive and non-conductive objects by shunting the electric field lines emerging from the sensor. As objects such as a human hand or a metal plate, which acts as a grounded conductor, approach the proximity sensor, the electric field lines from the upper electrode (fringe field) reach the object^41^. This decreases the electric field strength *E* of the capacitor of the proximity sensor, whereby the charge and capacitance of the proximity sensor decreases^40^. Based on this phenomenon, the proximity sensor can detect an approaching object.

To characterize the proximity sensing performance of the multifunctional sensor, the change in the voltage between the very bottom and topmost layers composed of non-aligned Ni Fs was measured as a steel plate (10 × 10 cm^2^) approached the top layer (cf. Figs. 1 and 2c). The initial distance between the steel plate and the sensor was 30 cm, and the initial variation voltage between the two electrodes in all cases was −2 V. From the initial position, the steel plate was allowed to approach the sensor (being parallel to it) to a close distance of 50 mm and was held for 10 s before being returned to its initial position. During this process, the voltage variation (*ΔV*_i_) between the two electrodes *versus* variation of the distance between the steel plate and the sensor (*d*_e_) was measured at distances of 70, 60, 50, 40, 30, 20, 10, 5, and 1 mm.

**Figure 4:**
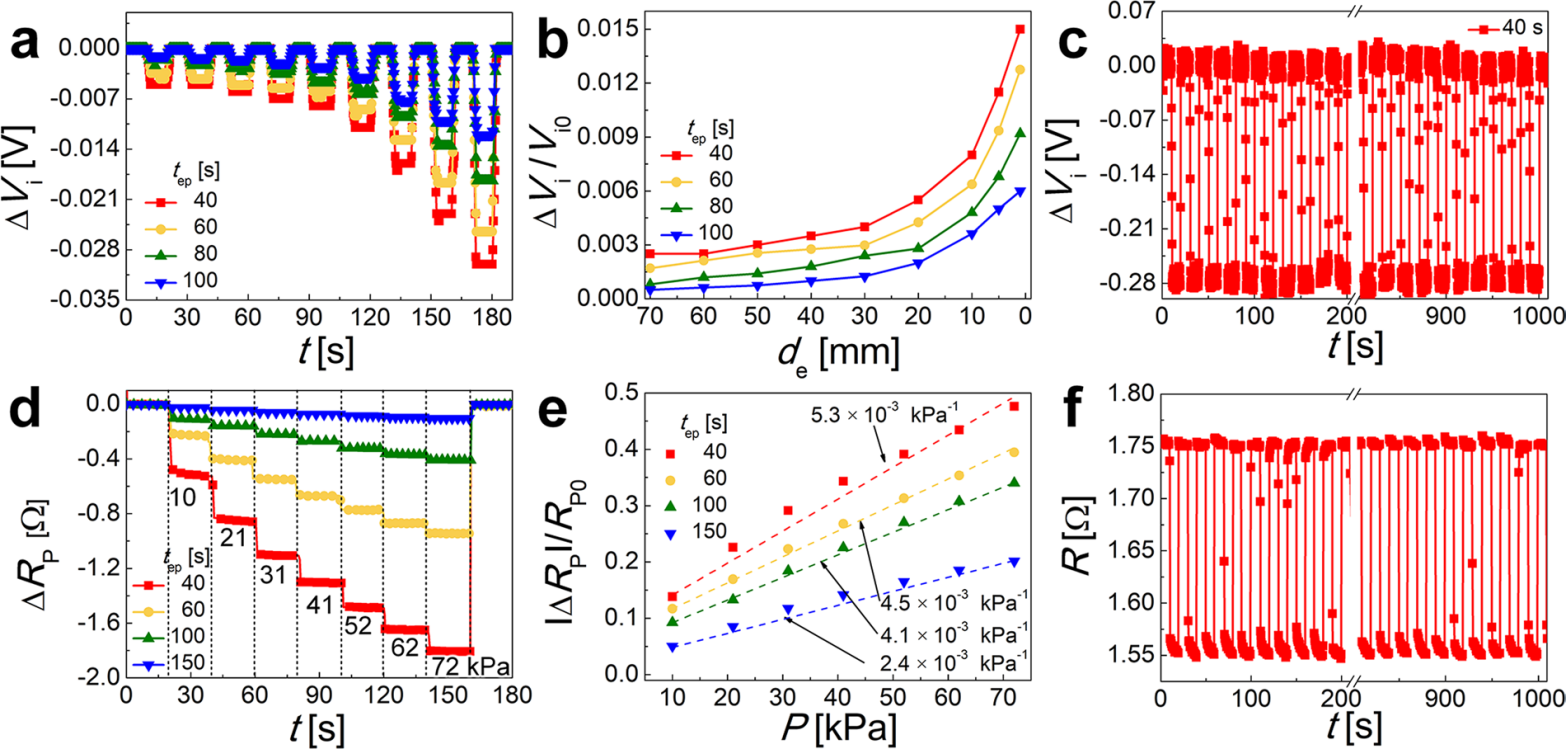
Repeatable performance of multifunctional sensor as proximity and pressure sensor: (**a**) Δ*V*_i_ for different values of *d*_e_ and different electroplating times *t*_*ep*_. (**b**) Δ*V*_i_/*V*_i0_ of the sensor as a function of *d*_e_. (**c**) Repeatable proximity sensing response for *d*_e_ = 5 mm in the case of *t*_ep_ = 40 s. (**d**) Δ*R*_P_ of different cases as a function of the applied weight. (**e**) |Δ*R*_P_|/*R*_P0_ of the sensor as a function of *P*. (**f**) Repeatable pressure sensing response at *P* = 41 kPa using the *t*_ep_ = 40 s sample.

As shown in Fig. 4a, in all cases, the negative *ΔV*_i_ increased in magnitude as the distance, *d*_e_, decreased, which can be attributed to the change in the capacitance *C*. According to Eq. (S6), |*ΔV*_i_| increases as the steel plate moves closely to the upper electrode of the sensor because |*Δd*_e_| increases; thus, the corresponding *d*_e_ can be determined. For the sensor employing the fibers prepared at *t*_ep_ = 40 s, when the steel plate was located 70 mm away from the upper electrode of the sensor, the value of *ΔV*_i_ was −0.005 V (red line in Fig. 4a). With a decrease in *d*_e_ to 60, 50, 40, 30, 20, 10, 5 and 1 mm, the value of *ΔV*_i_ (at *t*_ep_ = 40 s) increased to −0.005, −0.006, −0.007, −0.008, −0.011, −0.016, −0.023, and −0.03 V (red line in Fig. 4a), respectively. This behavior can be attributed to an increase in the variation in voltage (*ΔV*_e_) between the upper electrode of the sensor and the steel plate as *d*_e_ decreased, while *Δd*_e_ increased (cf. Fig. S3e). The detection limit of the proximity sensor is approximately *d*_e_ = 60 mm, beyond which proximity detection is difficult. As the electroplating time (*t*_ep_) increased to 60, 80, and 100 s, *ΔV*_i_ at *d*_e_ = 70 mm decreased to −0.0034, −0.0016, and −0.001 V, respectively. This tendency was observed for all values of *d*_e_, which is attributed to the surface area of the electrode according to Eq. (S6). In other words, when an equal charge (*Q*_e_) is applied to the two electrodes of the sensor at fixed values of *d*_e_ and *ε*_a_, *ΔV*_i_ decreases as the area (*A*) increases. As the electroplating time *t*_ep_ increased, *A* increased because of the increased diameter (*D*) of the Ni Fs (Table 1). As a result, the values of *ΔV*_i_ at *d*_e_ = 1 mm decreased from −0.03 to −0.025, −0.018, and −0.012 V as *t*_ep_ was increased, and thus, *A* increased.

Figure 4b shows the normalized variation of the potential difference (*ΔV*_i_/*V*_i0_) as a function of *d*_e_ for all cases. The sensor prepared at *t*_ep_ = 40 s exhibited the steepest slope for the plot of *ΔV*_i_/*V*_i0_ with decreasing *d*_e_. Note that the slope decreased as *t*_ep_ increased. To evaluate the reliability of the proximity sensing, the repeatability of the proximity response was explored using the sensor employing fibers prepared at *t*_ep_ = 40 s. The steel plate was allowed to approach the sensor to *d*_e_ = 5 mm and was maintained at this position for 10 s, after which the steel plate was returned to its initial position (30 cm). The process was repeated 50 times. As shown in Fig. 4c, the same value of *ΔV*_i_ of −0.024 V was obtained in all ten cases. Furthermore, the responses of the proximity sensor to a human palm and steel plate were confirmed. The voltage variation was measured using the proximity sensor at *t*_ep_ = 40 s, as the human palm approached from 50 to 2 mm. With a decrease in the distance between the upper electrode and the human palm from 50 to 2 mm, *ΔV*_i_ changed from −0.004 to −0.01, −0.013, and −0.018 V (cf. Fig. S3f). Accordingly, the proximity sensor can react to a human body in a similar way as with a steel plate, which is a conductor.

The multifunctional sensor can detect an applied pressure based on the variation in the resistance between the two electrodes, as depicted in Fig. 1c. To evaluate the pressure sensing performance, the resistance between the two perpendicularly stacked layers of aligned Ni Fs was measured as the weight placed on top of the sensor increased (cf. Fig. 1c), with the pressure applied to the sensor simultaneously measured by a pressure meter that was in contact with the bottom of the sensor. The applied mass was increased from 0.04 to 0.08, 0.12, 0.16, 0.2, 0.24, and 0.28 kg, corresponding to pressures of 10, 21, 31, 41, 52, 62, and 72 kPa, respectively, for the applied area of 3.8 × 10^−5^ m^2^. The detection limit of the pressure sensor is approximately 72 kPa, beyond which there was little difference in the resistance change. The time during which the weight was placed on the sensor and the interval between the tests was 20 s. Note that the initial resistances of the sensors (*R*_P0_) employing the fibers prepared with *t*_ep_ = 40, 60, 100, and 150 s were *R*_P0_ = 3.8, 2.5, 1.2, and 0.5 Ω, respectively.

The change in the resistance (Δ*R*_p_ = *R*_p_ − *R*_p0_) of the sensor is shown in Fig. 4d. In all cases, Δ*R*_p_ increased as pressure *P* increased because the half-width *a*_c_ of the contact zone increased as *P* increased (cf. Fig. S4a). For the sample formed with the electroplating time *t*_ep_ = 40 s, Δ*R*_p_ increased in magnitude with the following values: −0.5 to −0.9, −1.1, −1.3, −1.5, −1.7, and −1.8 Ω, with corresponding *P* values of 10, 21, 31, 41, 52, 62, and 72 kPa. However, when the pressure was fixed at *P* = 72 kPa, *ΔR*_p_ decreased in magnitude with the values −1.8 to −1.0, −0.4, and −0.1 Ω as the electroplating time *t*_ep_ increased from 40 to 60, 100, and 150 s, respectively.

This effect is attributed to the fact that *R*_p_ decreased as *D* or 2*r*_c_ increased for the Ni Fs at a fixed pressure. As illustrated in Fig. S4a, when two aligned Ni Fs come into contact, *a*_c_ and *P* from the Hertz solution for two cylinders in contact^43^ are related as

5 $${a}_{c}=\frac{4}{\pi }\frac{(1-{\nu }^{2})}{E}DP$$

where *D* is the fiber diameter, *E* is Young’s modulus, and *ν* is Poisson’s ratio of the fibers, and the applied pressure is related to the applied force per unit fiber length as *P* = *F*/2*a*_c_.

The latter yields *R*_p_ as:

6 $${R}_{{\rm{p}}}=\frac{\rho }{2{a}_{{\rm{c}}}}=\frac{C}{P},C=\frac{\rho \pi E}{8(1-{\nu }^{2})D}$$

Taking the logarithmic derivative of Eq. (6) yields:

7 $$\frac{1}{{R}_{{\rm{p}}}}\frac{\Delta {R}_{{\rm{P}}}}{\Delta P}=-\,\frac{1}{P}$$

Note that Eq. (7) implies that the product of *R*_p_*P* is a constant.

The response time *t*_r_ of the pressure sensor also varied with *t*_ep_ (Fig. S4b,c). When the weight was placed on top of the sensor, time *t*_r_ decreased from 1.8 to 1.41, 0.8, and 0.3 s as time *t*_ep_ increased from 40 to 60, 100, and 150 s, respectively (see Fig. S4b). When the weight was removed from the sensor, the times for recovery of the initial resistance value were *t*_r_ = 0.3, 0.7, 1.1, and 1.9 s for the sensors employing the fibers prepared with increasing time *t*_ep_ of 40, 60, 100, and 150 s, respectively (see Fig. S4c).

The variation of *ΔR*_p_ was more significant for pressure sensing in the case of fibers prepared with a short electroplating time *t*_ep_ (i.e., *t*_ep_ = 40 and 60 s) than for those with a higher *t*_ep_ (i.e., *t*_ep_ = 100 and 150 s). Similarly, the sensitivity of pressure sensing (*η*_p_ = |*ΔR*_p_|/(*R*_p0_*ΔP*) increased as time *t*_ep_ decreased (see Fig. 4e and Table 2). When increasing *P* from 10 to 72 kPa, the value of |*ΔR*_p_|/*R*_p0_ also increased from 0.138 to 0.476 for the sample formed at the electroplating time *t*_ep_ = 40 s. The value of *η*_P_ = 5.3 × 10^−3^ kPa^−1^ was the highest for this sample. At increasing *t*_ep_ to 60, 100, and 150 s, the value of |*ΔR*_p_|/*R*_p0_ decreased and the corresponding slope in the dependency on *P* (namely, *η*_p_) gradually decreased to 4.5, 4.1, and 2.4 × 10^−3^ kPa^−1^, respectively. The properties of the present pressure sensor are compared with the previously reported values in Table 3. The pressure sensing limit of the present sensor is wider than those previously reported.

**Table 3:** Properties of the pressure sensor compared with previous literature values.

Materials	Measured properties	Minimum sensitivity (kPa^−1^)	Maximum sensitivity (kPa^−1^)	*P* (kPa)	Reference
Carbon nanotube (CNT)	Resistance	0.005 (2 < *P* < 10 kPa)	0.12 (0 < *P* < 2 kPa)	2–10	^44^
Polypyrrole	Resistance	0.08 (0.1 < *P* < 11 kPa)	0.317 (0 < *P* < 0.1 kPa)	0–11	^45^
Polyethylene glycol diacrylate (PEGDA)/2-hydroxy-2-methylpropiophenone (HOMMP)/1-ethyl-3-methyl-imidazolium tricyanomethanide (EMIM/TCM)	Capacitance	0.12 (2.3 < *P* < 11.3 kPa)	0.55 (1 < *P* < 2.3 kPa)	1–11.3	^46^
Graphene oxide	Capacitance	0.005 (40 < *P* < 110 kPa)	0.96 (8 < *P* < 40 kPa)	8–110	^47^
Silver nanowire (AgNW)	Capacitance	0.88 (0.025 < *P* < 0.07 kPa)	5.54 (0 < *P* < 0.025 kPa)	0.025–0.07	^48^
Gallium (Ga)/Indium (In)	Voltage	0.08		0–50	^49^
Zinc oxide (ZnO)/Polystyrene	Current	3.4 (7 < *P* < 11.8 kPa)	10.3 (0 < *P* < 2 kPa)	0–11.8	^9^
Nickel fiber (Ni F)	**Resistance**	**0.0053**	**0**–**72**	**Present**	

Figure 4f illustrates the reliability of pressure sensing for the sensor employing the fibers formed with the electroplating time *t*_ep_ = 40 s, which exhibited the highest sensitivity (cf. Fig. 4e). The mass of 0.16 kg (equivalent to 1.56 N and 41 kPa over the area of 3.8 × 10^−5^ m^2^) was placed on the sensor and maintained for 10 s. The mass was then removed from the sensor (returning to zero force and pressure) and placed again after 10 s. This procedure was repeated *N* = 50 times. The same value of *ΔR*_p_ of −1.3 Ω was observed for each cycle of the test.

To evaluate the mechanical stability of the pressure sensing component of the multifunctional sensor and the ability for motion detection, the resistance was measured while the sensor with the fibers formed at *t*_ep_ = 40 s was repeatedly bent with a bending radius of 3 mm. The sensor was maintained for 5 s in the bent state and then allowed to recover to the original flat shape. The bending test was repeated ten times. As shown in Fig. S5a, the value of *ΔR*_p_ was approximately 0 Ω for the initial state (corresponding to the image in Fig. S5b), but increased in magnitude according to the value of −2.8 Ω when the sensor was bent, as shown in Fig. S5c. Then, the value of *ΔR* for the sensor was almost the same, −2.8 Ω, during the repeated bending, and returned to 0 Ω immediately when no pressure was applied. In addition, the performance of the pressure sensor at the bending radius of 20 mm was evaluated by applying pressure of 10 kPa to the bent pressure sensor 10 times (see Fig. S5d). As the pressure sensor was bent with a bending radius of 20 mm, the resistance of the pressure sensor decreased to 0.9 Ω, and the resistance of the bended pressure sensor further decreased by 0.12 Ω when a pressure of 10 kPa was applied 10 times during the cycle test.

### Active matrix pressure sensor and finger-tip multifunctional sensor

**Figure 5:**
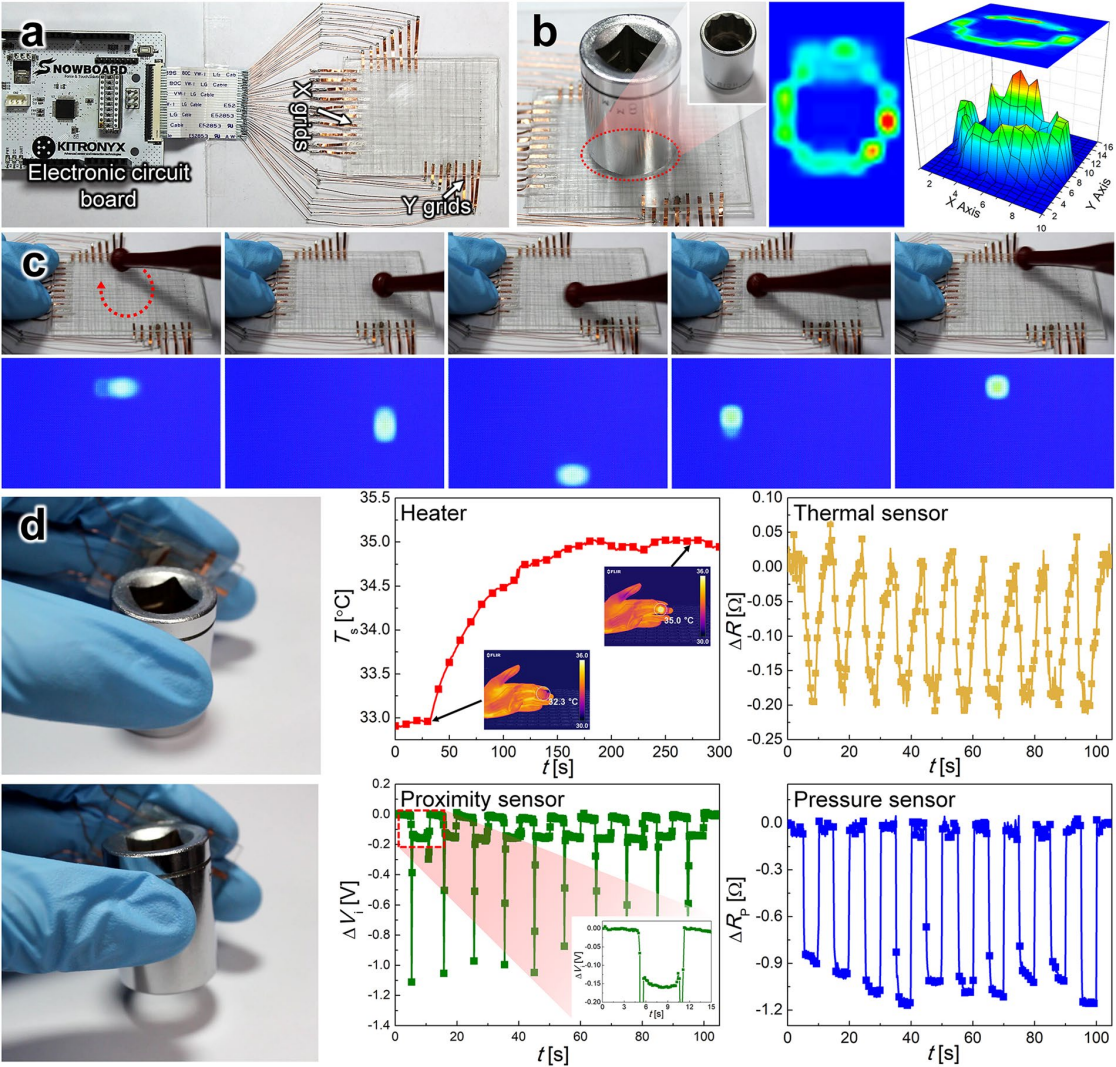
(**a**) Photograph of the active-matrix pressure sensor and the electric circuit board. (**b**) Images of the object with a doughnut-shaped base on top of the sensor and the corresponding visualization. (**c**) Photograph of the process of drawing a circle on the surface of the sensor and the corresponding visualization images. (**d**) Photograph of the finger-tip multifunctional sensor and the corresponding heating, as well as the thermal-, proximity-, and pressure-sensing performance.

In addition to the multifunctional sensor, an active matrix sensor (4.5 × 6 cm^2^ with 10 grids on the X-axis and 16 grids on the Y-axis, see Fig. 5) was developed using the aligned Ni Fs and an electronic circuit that can convert particular physical stimuli to digital signals. The time *t*_ep_ of the aligned Ni Fs used for the active matrix pressure sensor was *t*_ep_ = 40 s, thus allowing the sensor to be highly transparent (cf. Fig. 5a–c). When an object with a doughnut-shaped base was placed on the surface of the active matrix pressure sensor, as shown in Fig. 5b, a visualized pressure image and the corresponding 3D plot were clearly presented on the screen with a shape identical to that of the bottom of the object. In addition, a circle was drawn in a clockwise direction on the sensor, and the corresponding pressure pattern was correctly detected along the drawing path. Note that the writing of characters could also be detected by the sensor, as confirmed through the visualized pressure images of the sensor (see Movie S1).

A finger-size (1.5 × 1.5 cm^2^) multifunctional sensor was fabricated using the non-aligned Ni Fs formed with *t*_ep_ = 100 s for heating, and thermal and proximity sensing, in conjunction with the aligned Ni Fs formed with *t*_ep_ = 150 s used for pressure sensing. By attaching the sensor between the forefinger and the middle finger and holding an object and repeatedly lifting it, as shown in Fig. 5d, one can confirm simultaneous operation of the three functions of the sensor, where a voltage was also applied to bring the temperature close to that of the human body.

Prior to holding the object, a voltage of 0.4 V (corresponding to 0.155 W) was applied to the heater located in the bottom layer of the multifunctional sensor. Note that the initial temperature of the multifunctional sensor was 33.0 °C. The temperature of the sensor increased immediately after the application of voltage to the heater, reaching 35.0 °C in 170 s. This temperature was maintained for up to 300 s.

While maintaining the temperature of the multifunctional sensor at 35.0 °C, the object was held, lifted for 5 s, and placed again at its initial position. This procedure was repeated ten times. As shown in Fig. 5d, the resistance change with respect to the temperature change is highly responsive and sensitive. The temperature change of the object is related to the sensor resistance change (cf. Fig. 5d), which is highly repeatable and responsive.

When the sensor on the fingers approached an object at close proximity, the potential difference *ΔV*, associated with proximity sensing, increased dramatically in magnitude and reached the value of −1.1 V, as shown in Fig. 5d. While holding and lifting the object, *ΔV* was maintained at −0.15 V. Note that the reliability of the finger-tip sensor for proximity sensing was confirmed in ten repeated tests.

Moreover, *ΔR*_p_, associated with pressure sensing, decreased when the object was held or lifted. Because the force applied to hold the object varied slightly, the corresponding *ΔR*_p_ decreased to a slightly different level in each case. However, *ΔR*_p_ immediately recovered to its initial value once the object was placed down.

As demonstrated in Fig. 5d, the finger-tip multifunctional sensor can simultaneously detect temperature change, an approaching object, and an applied pressure while maintaining the temperature at 35 °C (Movie S2). The present multifunctional sensor consists of four layers with non-aligned and aligned Ni Fs in one cell, with each layer featuring each unique sensor capability. Readings of each layer can be done fully independently, selecting and separating one of the four sensor functions on demand. In other words, this multifunctional sensor, which is akin to the structure of the human skin, holds great promise as a next-generation material for anthropomorphic robots.

## Conclusion

Inspired by the cutaneous sensing system of the human skin, multifunctional sensors capable of thermal, pressure, and proximity sensing, as well as heating, were fabricated using non-aligned and aligned Ni Fs for potential use as artificial electronic skin (e-skin). The fabrication methods introduced in the present work are fast, inexpensive, and straightforward, thus making fabrication economically viable. The fabrication process was optimized to produce transparent, flexible sensors with multifunctionality. The developed multifunctional sensors provide features such as a low sheet resistance, high transparency, and flexibility, highlighting their potential for use as transparent electronic skin.

## Supplementary information

Supplementary Information.

Movie 1.

Movie 2.
