# Supplementary material for: Transparent Body-Attachable Multifunctional Pressure, Thermal, and Proximity Sensor and Heater
Source: Sci Rep. 2020 Feb 14;10:2701. doi: 10.1038/s41598-020-59450-0 (PMC7021770; doi:10.1038/s41598-020-59450-0)
Supplement: Supplementary file 1 — Supplementary Information. [file 41598_2020_59450_MOESM1_ESM.pdf]

Transparent Body-Attachable Multifunctional Pressure, Thermal, and Proximity Sensor and Heater

Hong Seok Jo[a,†], Seongpil An[b,†,*], Hyuk-Jin Kwon[a], Alexander L. Yarin[c,*], Sam S. Yoon[a,*]

[a]School of Mechanical Engineering, Korea University, Seoul 02841, Republic of Korea
[b]SKKU Advanced Institute of Nanotechnology (SAINT) Department of Nano Engineering Sungkyunkwan University (SKKU) Suwon 16419, Republic of Korea
[c]Department of Mechanical and Industrial Engineering, University of Illinois at Chicago, 842 W. Taylor Street, Chicago, Illinois 60607-7022, United States

***Corresponding author:** esan@skku.edu, ayarin@uic.edu, skyoon@korea.ac.kr

†These authors have equally contributed

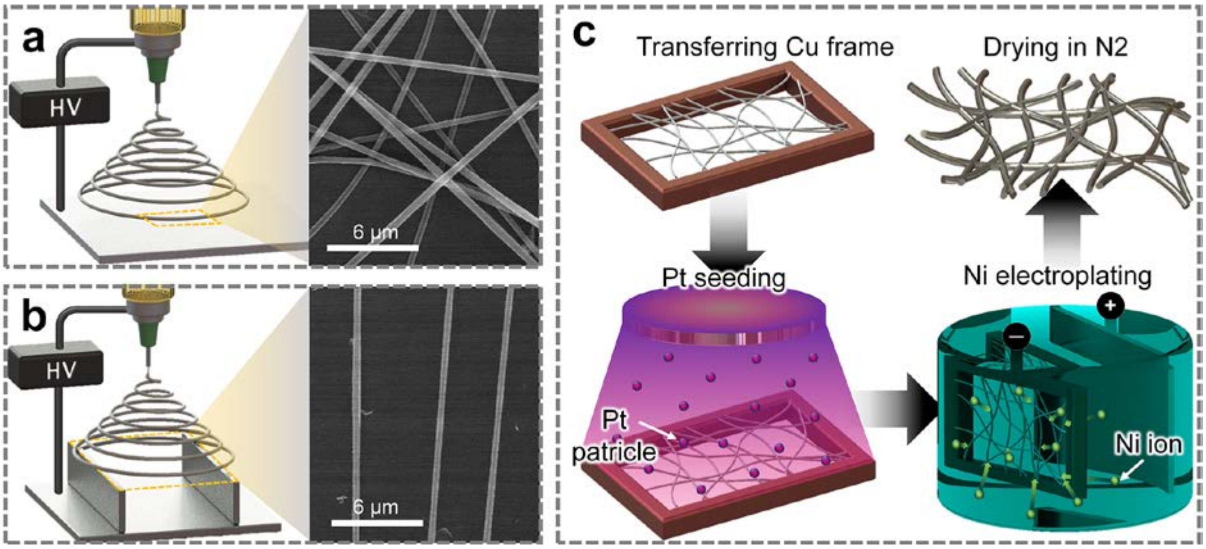

Figure S1. Schematic of the fabrication processes for (a) the non-aligned, (b) the aligned PAN, and (c) the Ni Fs by electroplating.

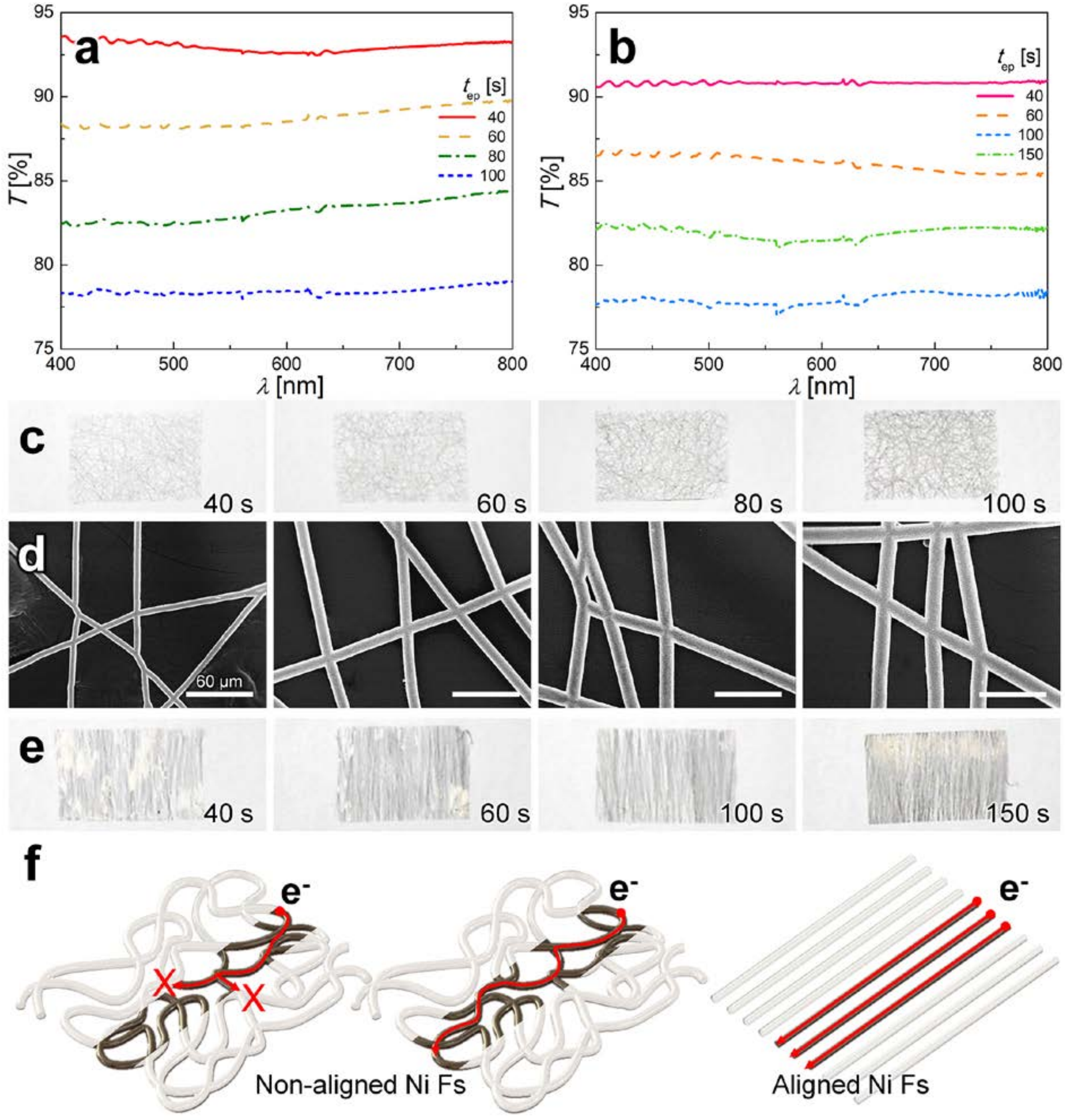

Figure S2. Transmittance of (a) the non-aligned and (b) aligned Ni Fs with various t_{ep}. (c) Photos and (d) SEM images of the non-aligned Ni Fs as a function of t_{ep}. (e) Photos of the aligned Ni Fs as a function of t_{ep}. (f) Illustration of the electron mobility of the non-aligned and aligned Ni Fs when the edges of fibers were cut.

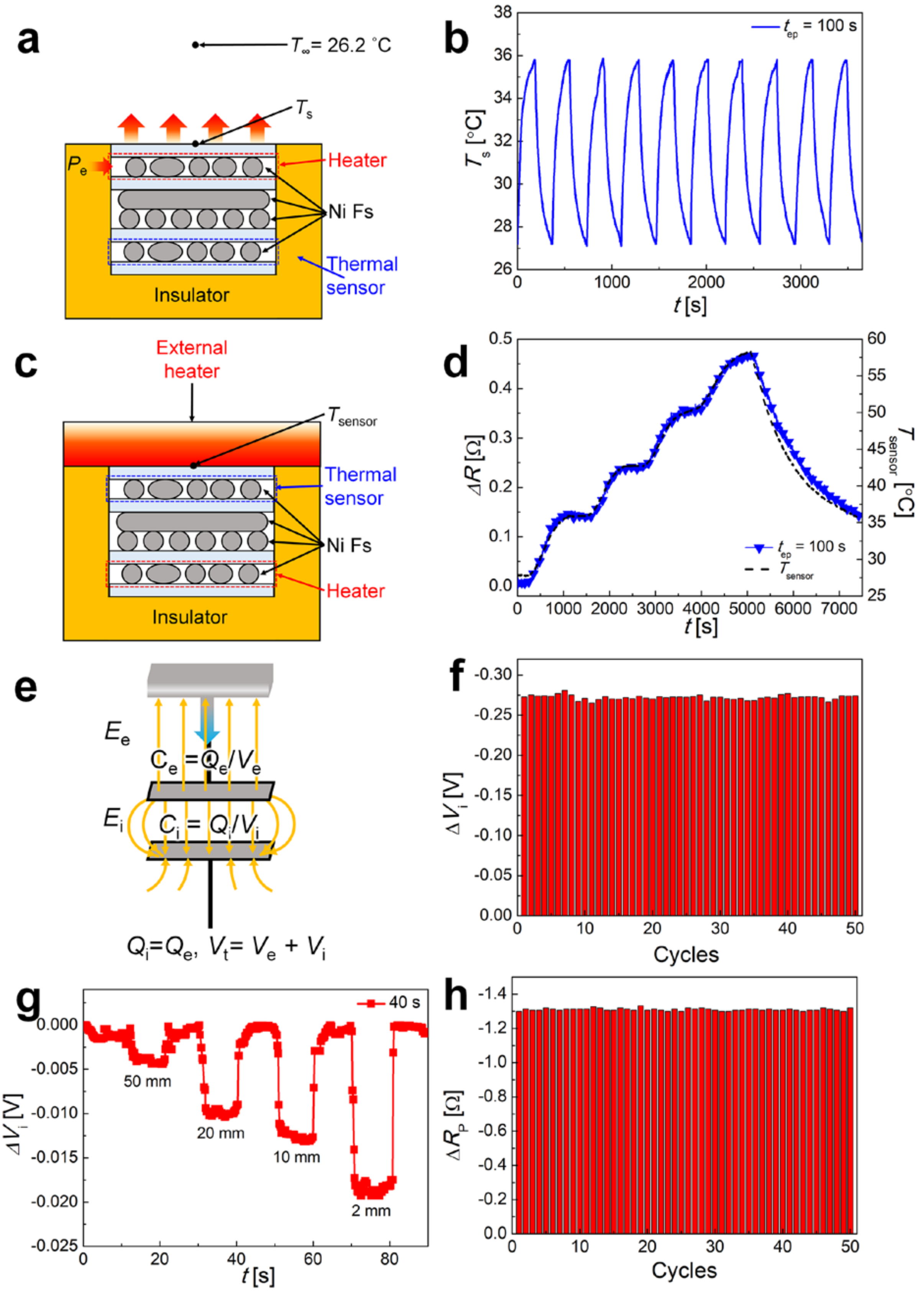

Figure S3. (a) Measurement method for heater and (b) cyclic rise and fall of the heater

temperature under the supplied voltage of 0.3 V using the sensor sample with t_{ep} = 100 s. (c) Measurement methods for thermal sensor. (d) Resistance change of the temperature sensor over 7500 s. (e) The working mechanism of the multifunctional sensor as a proximity sensor. E_i is the electrical field between two electrodes in the sensor (cf. **Figure 1**e). E_e is the electric field between the upper electrode of the sensor and the approaching steel plate. (f) The cycle was repeated 50 times, and (g) the response of the proximity sensor with t_{ep} = 40 s to a human palm. (h) The pressure change cycle was repeated 50 times.

The effective heat transfer coefficient h_m can be described as:

$$h_m = \frac{1}{\Delta x_s / k_s + \Delta x_i / k_i + 1/h_b} \tag{S1}$$

where Δx_s and k_s are the overall thickness and thermal conductivity, respectively, of the material between the heater and the bottom-most insulator (cf. **Figure S3**a); Δx_i and k_i are the thickness and thermal conductivity of the bottom-most insulator, respectively (cf. **Figure S3**a); and h_b is the convective heat transfer coefficient at the surface of the bottommost insulator (cf. **Figure S3**a).

The Δx_s consists of 3 layers of the Ni Fs (where the total thickness Δx_{Ni} and thermal conductivity k_{Ni} of the Ni Fs are 15 μm and 90 $W \cdot m^{-1} \cdot °C^{-1}$, respectively). Therefore, $\Delta x_s/k_s$ can be expressed as $\Delta x_s/k_s = 3\Delta x_{Ni}/k_{Ni}$, and the resulting value is 5×10^{-7} $°C \cdot m^2 \cdot W^{-1}$. The value of $\Delta x_i/k_i$ is 0.5714 $°C \cdot m^2 \cdot W^{-1}$ for Δx_i = 0.02 m and k_i = 0.035 $W \cdot m^{-1} \cdot °C^{-1}$.

The value of h_b can be obtained using its dimensionless equivalent, the Nusselt number Nu, as $h_b = k_{air} \cdot Nu/L_c$, where k_{air}, Nu, and L_c are the thermal conductivity of air at T_{eff} (0.025 $W \cdot m^{-1} \cdot °C^{-1}$), the Nusselt number, and the side length of the plate (0.005 m), respectively. Here, the effective surface temperature T_{eff} is defined as $T_{eff} = (T_s+T_\infty)/2$. To determine the value of

Nu, the Rayleigh number is first found as Ra = 12.03 from its expression: Ra = $g\beta(T_s - T_\infty)L_c^3\mathrm{Pr}/\nu^2$, where g is the acceleration of gravity; Pr and ν are the Prandtl number and the kinematic viscosity of air at T_{eff}, respectively (Pr = 0.7290 and $\nu = 1.575\cdot10^{-5}$ m^2/s), and β is the average volumetric thermal expansion coefficient $\beta = 1/T_{eff}$. Then, the value of Nu can be found using the expression Nu = $C\cdot\mathrm{Ra}^m$, where C and m are known parameters. When the value of Ra is in the range of $10 < \mathrm{Ra} < 2.5 \times 10^2$, C and m are 0.906 and 0.089, respectively,[1] and thus, the value of Nu is 1.13; accordingly, the value of h_b is 5.804 W·m^{-2}·°C^{-1}.[2]

As a result, h_m can be obtained from **Eq. (S1)** as 0.0001345 W·cm^{-2}·°C^{-1}, which seemingly does not significantly affect the value of h of the heater because of the relatively lower value of h_m [cf. **Eq. (3)**].

We define the capacitance between the upper electrode and the steel plate as C_e (see **Fig. S3**e and note that $C_e = \varepsilon_a A/d_e = Q_e/V_e$. Here, ε_a, A, Q_e, and V_e are the dielectric permittivity of air, the surface area of the electrode, the storage charge, and the voltage between the upper electrode and the steel plate, respectively. Also, the capacitance between two electrodes of the proximity sensor is C_i (see **Fig. S3**e and note that $C_i = \varepsilon_i A/d_i = Q_i/V_i$, where ε_i, Q_i, d_i, and V_i are the dielectric permittivity of PDMS, the storage charge, the distance between the two electrodes, and the voltage between two electrodes, respectively[3]). Because of the series connection of the C_e and C_i, the values of Q_e and Q_i are equal and the total voltage V_t (which is the sum of V_e and V_i) is constant at −2 V. Note that the initial capacitance is estimated to be approximately $C_{theory} = I/(2\pi\cdot f\cdot V) = 0.79$ nF, where $I = 10$ μA, $f = 1$ kHz is the resonance frequency, and $V = 2$ V. On the other hand, the measured capacitance was $C_{exp} = 0.75$ nF. The theoretical and experimental values are close enough to confirm their validity. In addition, the quality factor of the proximity sensor, which is expressed as $1/fCR$, was 0.6 at R =2.2 MΩ connected with the proximity sensor.

The inverse of the total capacitance is $1/C_t = 1/C_e + 1/C_i$. It can also be written as:

$$C_{t} = \frac{C_{e}C_{i}}{C_{e}+C_{i}} = \frac{a}{1+b\cdot d_{e}},\ \text{where } a = \frac{\varepsilon_{i}A}{d_{i}} \text{ and } b = \frac{\varepsilon_{i}}{\varepsilon_{a}d_{i}} \tag{S2}$$

In a series connection,

$$Q_{t} = Q_{e} = Q_{i} = C_{t}V_{t} = \frac{a}{1+b\cdot d_{e}}V_{t} \tag{S3}$$

Therefore, as the steel plate approached the upper electrode of the sensor, corresponding to a decrease in d_e, both Q_e and Q_i increased. In addition, the potentials are expressed as:

$$V_{e} = \frac{d_{e}}{\varepsilon_{a}A}Q_{e} = \frac{d_{e}}{\varepsilon_{a}A}\left(\frac{a}{1+b\cdot d_{e}}V_{t}\right) = \frac{V_{t}}{\varepsilon_{a}A}\left(\frac{a}{1/d_{e}+b}\right) \tag{S4}$$

$$V_{i} = \frac{d_{i}}{\varepsilon_{i}A}Q_{i} = \frac{d_{i}}{\varepsilon_{i}A}\left(\frac{a}{1+b\cdot d_{e}}V_{t}\right) \tag{S5}$$

Thus, when d_e decreases, V_e decreases and V_i increases. Also, assuming V_t is constant at – 2 V, as confirmed by a source meter, the variation of V_i can be calculated with respect to d_e as:

$$\begin{aligned} V_{i} &= V_{t} - V_{e} \\ \Delta V_{i} &= \Delta V_{t} - \Delta V_{e} \\ \Delta V_{i} &= -\Delta V_{e} = -\frac{Q_{e}}{\varepsilon_{a}A}\Delta d_{e} \end{aligned} \tag{S6}$$

Initially, $V_e = 0$, and thus, $V_{i0} = V_t$; see **Fig. S3**c.

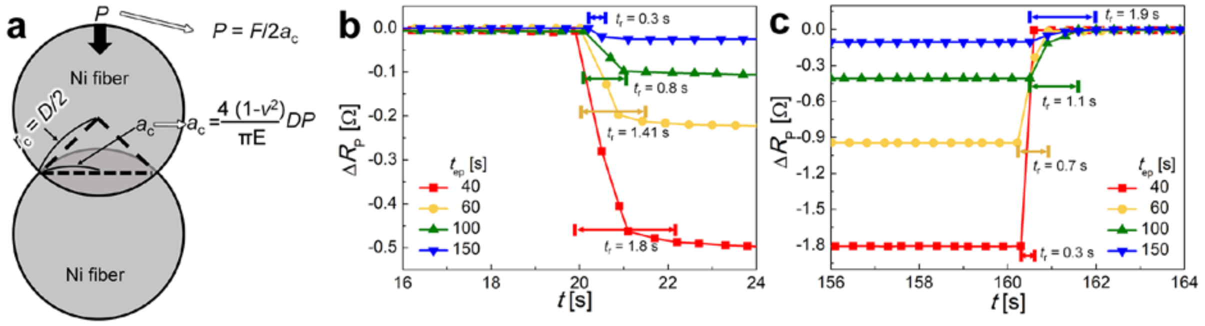

Figure S4. (a) Sketch of the underlying mechanism for pressure sensing and (b, c) the corresponding response speed (b) when the object is loaded and (c) when the object is removed.

When the applied pressure increases, the contact resistance between the two perpendicularly stacked layers of the aligned Ni Fs decreases because the contact area between the layers increases (cf. **Fig. S4**a). This phenomenon can be described by the electrical constriction resistance (R_{p}) as [4,5]:

$$R_{\mathrm{p}} = \frac{\rho}{2a_{\mathrm{c}}}\left[1 - 1.42\left(\frac{a_{\mathrm{c}}}{r_{\mathrm{c}}}\right) + 0.063\left(\frac{a_{\mathrm{c}}}{r_{\mathrm{c}}}\right)^{2} + 0.153\left(\frac{a_{\mathrm{c}}}{r_{\mathrm{c}}}\right)^{3} + 0.2\left(\frac{a_{\mathrm{c}}}{r_{\mathrm{c}}}\right)^{4}\right] \qquad \text{(S7)}$$

where ρ, a_{c}, and r_{c} are the electrical resistivity, the half-width of the contact zone, and the radius of the cylindrical conductor, respectively, as depicted in **Fig. S4**a. When $a_{\mathrm{c}} \ll r_{\mathrm{c}}$, the ratio ($a_{\mathrm{c}}/r_{\mathrm{c}}$) is negligibly small; thus, **Eq. (S7)** reduces to $R_{\mathrm{p}} = \rho/2a_{\mathrm{c}}$, where R_{p} decreases as a_{c} increases. Hence, the applied pressure can be detected through the change in the resistance between the two perpendicularly stacked layers of aligned Ni Fs in the sensor.

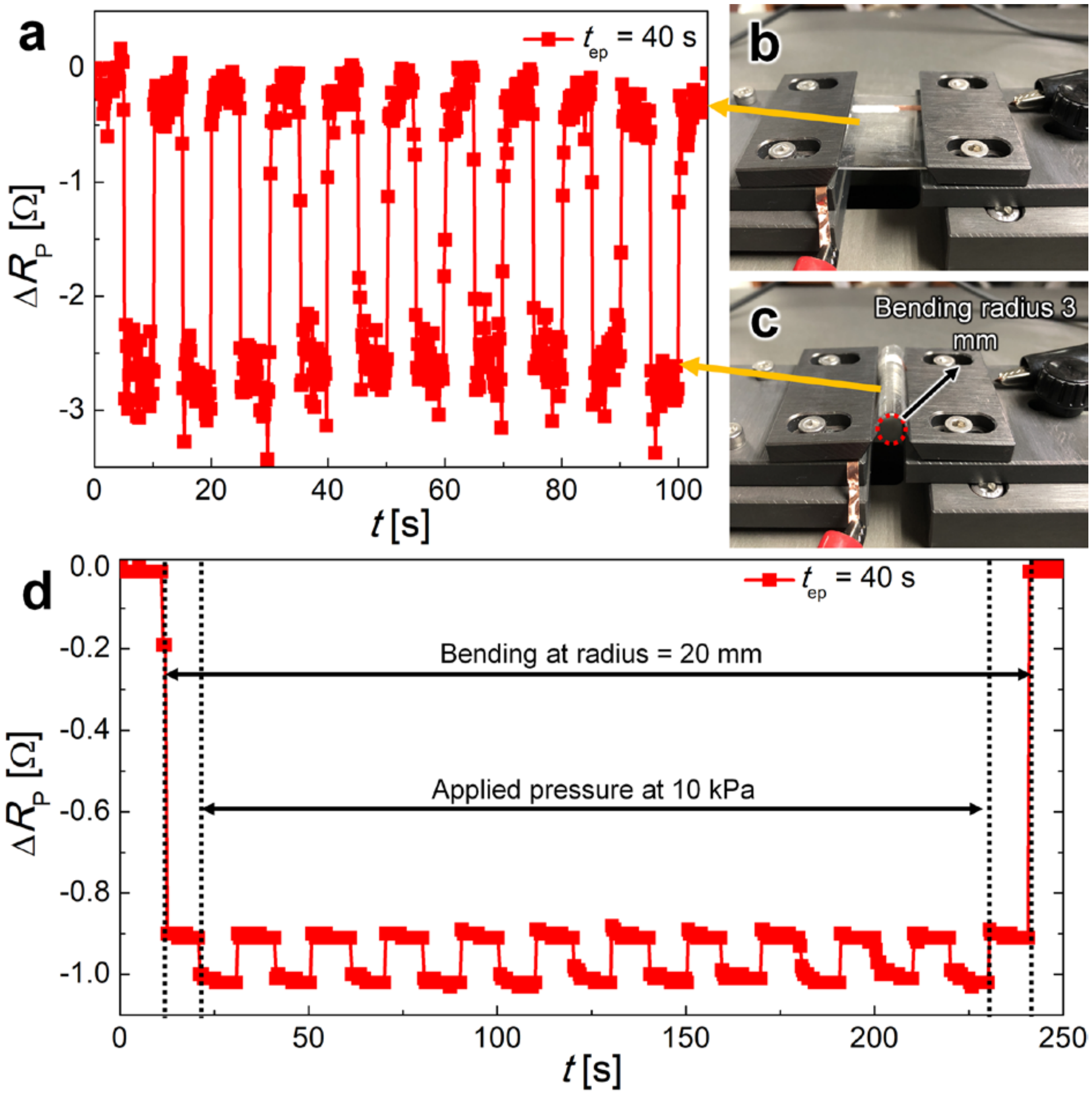

Figure S5. (a) Bending test of the pressure sensor of the multifunctional sensor and its corresponding photos at (b) the initial state and (c) the bending state. (d) Repeatable pressure sensing under a bending radius of 20 mm at P = 10 kPa.

References

1. Goldstein, R. & Lau, K.-S. Laminar natural convection from a horizontal plate and the influence of plate-edge extensions. *J. Fluid Mech.* **129**, 55-75 (1983).
2. Radziemska, E. & Lewandowski, W. Heat transfer by natural convection from an isothermal downward-facing round plate in unlimited space. *Appl. Energy* **68**, 347-366 (2001).
3. Halliday, D., Resnick, R. & Walker, J. *Fundamentals of Physics*. (John Wiley & Sons, 2013).
4. Kogut, L. & Komvopoulos, K. Electrical contact resistance theory for conductive rough surfaces. *J. Appl. Phys.* **94**, 3153-3162 (2003).
5. Timsit, S. in *Electrical Contacts-1998. Proceedings of the Forty-Fourth IEEE Holm Conference on Electrical Contacts (Cat. No. 98CB36238).* 1-19 (IEEE).
